# Longitudinal association of executive function and structural network controllability in the aging brain

**DOI:** 10.1007/s11357-022-00676-3

**Published:** 2022-10-21

**Authors:** Rongxiang Tang, Jeremy A. Elman, Carol E. Franz, Anders M. Dale, Lisa T. Eyler, Christine Fennema-Notestine, Donald J. Hagler, Michael J. Lyons, Matthew S. Panizzon, Olivia K. Puckett, William S. Kremen

**Affiliations:** 1 Department of Psychiatry, University of California San Diego, La Jolla, CA 92093 USA; 2 Center for Behavior Genetics of Aging, University of California San Diego, 9500 Gilman Drive, La Jolla, CA 92093 USA; 3 Department of Radiology, University of California San Diego, La Jolla, CA 92093 USA; 4 Department of Neurosciences, University of California San Diego, La Jolla, CA 92093 USA; 5 Desert Pacific Mental Illness Research Education and Clinical Center, VA San Diego Healthcare System, San Diego, CA 92093 USA; 6 Department of Psychological and Brain Sciences, Boston University, Boston, MA 02212 USA

**Keywords:** Executive function, Cognitive aging, Structural network, Controllability

## Abstract

Executive function encompasses effortful cognitive processes that are particularly susceptible to aging. Functional brain networks supporting executive function—such as the frontoparietal control network and the multiple demand system—have been extensively investigated. However, it remains unclear how structural networks facilitate and constrain the dynamics of functional networks to contribute to aging-related executive function declines. We examined whether changes in structural network modal controllability—a network’s ability to facilitate effortful brain state transitions that support cognitive functions—are associated with changes in executive function cross-sectionally and longitudinally. Diffusion-weighted imaging and neuropsychological testing were conducted at two time points (Time 1: ages 56 to 66, *N* = 172; Time 2: ages 61 to 70, *N* = 267) in community-dwelling men from the Vietnam Era Twin Study of Aging. An executive function factor score was computed from six neuropsychological tasks. Structural networks constructed from white matter connectivity were used to estimate modal controllability in control network and multiple demand system. We showed that higher modal controllability in control network and multiple demand system was associated with better executive function at Time 2, after controlling for age, young adult general cognitive ability, and physical health status. Moreover, changes in executive function over a period of 5 to 6 years (Time 1-Time 2, *N* = 105) were associated with changes in modal controllability of the multiple demand system and weakly in the control network over the same time period. These findings suggest that changes in the ability of structural brain networks in facilitating effortful brain state transitions may be a key neural mechanism underlying aging-related executive function declines and cognitive aging.

**Supplementary information:**

The online version contains supplementary material available at 10.1007/s11357-022-00676-3.

## Introduction

Executive functions encompass top-down cognitive processes necessary for goal-directed behavior and flexible adaptation to everyday life [1, 2]. Carrying out executive functions is fundamentally effortful, as it engages processes needed when “going on automatic or relying on instinct” would be insufficient to meet the current goals or demands [1]. It is well known that these functions are susceptible to aging, but the neural mechanisms underlying aging-related declines in executive function are not well understood. Recent advances in cognitive and network neuroscience suggest that higher order cognitive processes, including those involved in executive function, emerge from dynamic interactions among large-scale brain functional networks that are both facilitated and constrained by the underlying structural connectome [3, 4]. Consequently, delineating how the structural connectome contributes to aging-related declines in executive function would enrich our fundamental understanding of the neural mechanisms underlying cognitive aging and aging-related disorders that manifest executive dysfunction [5]. Such knowledge is important for developing and implementing treatment strategies to better target aging-related impairment in executive function.

**Fig. 1:**
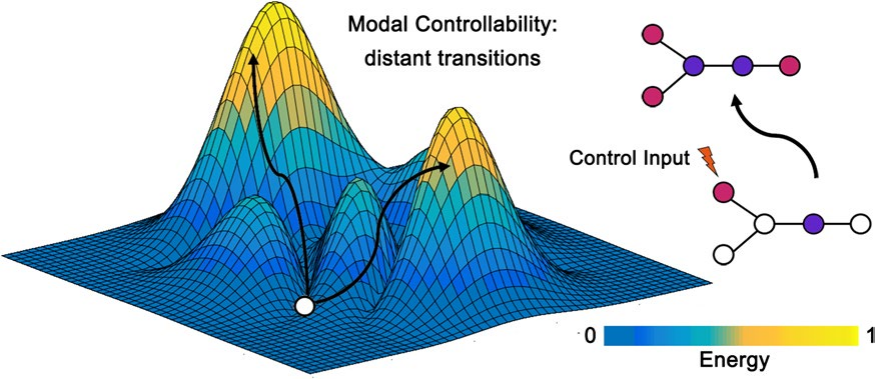
A schematic representation of modal controllability. In the left contour plot, the white dot represents a brain region facilitating distant transitions from the current mental state to difficult-to-reach mental states that require a considerable amount of input energy/effort as indicated by the darker to brighter color bar. The arrows represent control trajectories. The dot and line plots on the right depict a change in mental state from one to another after brain region(s) receive an input of control energy and initiate the transition

Leveraging recent theoretical and empirical advances in network neuroscience [3, 5, 6], we postulated that individual differences in executive function and changes in executive function over time in older adults can be explained by the controllability of structural brain networks. Controllability is a brain structural metric derived from white matter connectivity that quantifies the ease by which brain regions facilitate transitions between diverse mental states to enable complex cognitive processes [3]. Conceptually, controllability builds upon the notion that certain regions within the structural networks act as critical drivers of brain functional network dynamics, thereby controlling downstream cognitive processes and functioning [3, 7]. Indeed, studies on network controllability have demonstrated its association with cognitive performance in tasks tapping core executive functions (e.g., working memory, inhibition, cognitive flexibility) in children, adolescents, and young adults [8–10], indicating the cognitive relevance of network controllability across development and into young adulthood. One type of network controllability, modal controllability, quantifies brain regions that facilitate distant transitions to *difficult-to-reach* mental states that require substantial input energy or cognitive effort (Fig. 1) [3, 8]. The emphasis on effortful state transitions bears strong similarity to the definition of executive functions as being effortful cognitive processes [1], rendering modal controllability as a putative neural substrate of executive function.

Whether this association between structural network controllability and cognition persists through later adulthood to influence cognitive aging remains unexplored. Here, we examined the hypothesis that modal controllability is a predictor of executive function and its aging-related decline over time, aiming to provide a mechanistic explanation of how changes in structural properties of the brain translate into cognitive decline in older adults.

Prior work has illustrated that weakly connected brain regions within certain functional networks (i.e., frontoparietal, cingulo-opercular) exhibit high modal controllability, suggesting that they are responsible for the brain’s dynamics and transitions during effortful and difficult tasks [3, 11]. Moreover, decades of cognitive neuroscience research have demonstrated that these same functional networks are activated to support a diverse range of cognitive and executive control processes, particularly when there is increased task difficulty or demand that requires substantial cognitive effort [12–14]. Therefore, we focus our investigation of modal controllability specifically within the following two networks in the present study: the multiple demand system and the frontoparietal control network. The multiple demand system encompasses a set of key regions from the frontoparietal control network and salience/cingulo-opercular network and is associated with domain-general core cognitive processes [12, 15] as well as individual differences in intelligence [15, 16]. In particular, the multiple demand system is consistently activated in more cognitively demanding conditions (e.g., higher working memory load > low working memory load) that requires greater cognitive control, irrespective of the task domains (i.e., domain-general) [16]. The frontoparietal control network, or simply, the control network, was defined by the Schaefer functional network atlas based on large-scale task and resting-state fMRI data acquired using diverse acquisition protocols [17]. The control network includes frontal and parietal brain regions implicated in cognitive control processes and performance of executive functions tasks [18, 19] as well as cingulate cortex and temporal gyrus that are associated with control-related processes [17]. Although all frontal and parietal regions within the multiple demand system are included in the control network, the key distinction between the two networks is that the multiple demand system also includes regions from the salience/cingulo-opercular network implicated in response inhibition and maintenance of tonic alertness (i.e., sustained process of ensuring engagement) [20]. Notably, these salience/cingulo-opercular regions exhibit consistent neural activation in response to demanding task conditions along with the set of frontoparietal regions included in the multiple demand system [15, 16].

In the present study, we utilized diffusion-weighted imaging and six neuropsychological tasks from older adults at two different assessments conducted 5–6 years apart to examine the associations between modal controllability and executive function both cross-sectionally and longitudinally. Applying the framework of the well-established executive function model proposed by Miyake and Friedman [21, 22], these six neuropsychological tasks (Stroop, Trail Making Test, Category Switching, Letter-Number Sequencing, Reading Span, and Digit Span) were specifically chosen from our clinical neuropsychological task battery given they are the most relevant tasks tapping into classic executive function subdomains including inhibitory control, set shifting, and working memory. The Miyake-Friedman model provides strong evidence that these subdomains are well represented by a common executive function factor [21, 22], as used in the present study. Our prior work showed a small cross-sectional association between age and performance on these neuropsychological tasks (likely due to the narrow age range of our sample — 10 years), but steady longitudinal within-person decline in performance across time, which is consistent with most observations of age-related declines in executive function [23, 24]. Moreover, these neuropsychological tasks of executive function actively engage cognitive control processes (often considered synonymous with executive functions within the context of clinical neuropsychology [25]) to support the maintenance of task goals and the flexible regulation of one’s behavior and actions in accordance with those task goals [25].

For the formal analyses, we first computed modal controllability across all 400 Schaefer cortical parcels to characterize regional modal controllability at the group level. Second, we examined the associations of modal controllability in the control network and multiple demand system cross-sectionally at each time point. Third, we investigated whether longitudinal changes in modal controllability for each of the two networks were associated with changes in executive function, establishing a mechanistic explanation linking structure to function. Finally, we explored whether the link between structure and function was moderated by other factors: (1) *APOE* genotype (ε4 + vs. ε4 −), the major genetic risk factor for Alzheimer’s disease [26], and (2) general cognitive ability in young adulthood, an index of cognitive reserve [27–30]. If an interaction shows a weaker association between modal controllability and executive function in ε4 − or high general cognitive ability individuals compared to their respective counterparts, it would be consistent with the notion that those factors confer greater resilience against reduced modal controllability in older adults.

## Methods

### Participants

Participants were in the Vietnam Era Twin Study of Aging (VETSA), a multisite longitudinal study of aging and risk for Alzheimer’s disease beginning in middle age [31–33]. They are community-dwelling men from across the USA who are similar to American men in their age range with respect to health, education, and lifestyle characteristics [34]. VETSA has completed three waves of assessment. For the present study, we included participants who completed both neuroimaging and cognitive testing at wave 2 (ages 56 to 66) and wave 3 (ages 61 to 70) at the University of California, San Diego (UCSD) site on 3.0 Tesla scanners. All imaging was conducted at UCSD during wave 3, and we included only wave 2 participants who were scanned at UCSD to reduce scanner differences. Wave 1 data were not included because the imaging was collected on a 1.5 Tesla scanner, so they could not provide comparable estimates of structural network controllability. Here, we refer to wave 2 and wave 3 as Time 1 and Time 2 because they were the only two time points included in this report.

Participants were excluded for seizure, stroke, multiple sclerosis, HIV/AIDS, or schizophrenia. We further excluded participants with poor fiber tractography quality after our visual inspection (see Supplementary Methods for details). A total of 172 participants were included for Time 1, 267 participants for Time 2, and 105 participants for both time points. Table 1 contains a summary of sample characteristics. The study was approved by the Institutional Review Board at UCSD and Boston University, and written informed consent was obtained from all participants.

**Table 1:** Sample characteristics at each time point

	Time 1 (*N* = 172)	Time 2 (*N* = 267)	Longitudinal (*N* = 105)	
			*Time 1*	*Time 2*
Age	61.75 (2.69)	67.48 (2.73)	61.54 (2.72)	67.22 (2.76)
Years of education	13.98 (2.06)	14.14 (2.16)	14.02 (2.14)	
Young adult general^a^ cognitive ability (z-scores)	0.36 (0.65)	0.39 (0.67)	0.35 (0.61)	
Young adult general^a^ cognitive ability percentile	61.89 (21.61)	62.74 (21.60)	61.51 (20.17)	
Health status	1.07 (0.92)	1.35 (1.02)	0.99 (0.93)	1.25 (0.99)
*Race/ethnicity*				
(% White/Non-Hispanic) (% others)	87% (150) 13% (22)	89% (237) 11% (27)	90% (95) 10% (10)	
Executive function^b^	− 0.44 (0.94)	− 0.72 (0.92)	− 0.50 (0.97)	− 0.79 (0.94)
Multiple demand system modal controllability	0.94 (0.02)	0.94 (0.02)	0.94 (0.02)	0.94 (0.02)
Control network modal controllability	0.95 (0.02)	0.94 (0.02)	0.95 (0.02)	0.94 (0.02)
	Time 1 (*N* = 172)	Time 2 (*N* = 238)	Longitudinal (*N* = 105)	
*APOE-ε4 status*				
Positive Negative	42 (24%) 130 (76%)	60 (25%) 178 (75%)	25 (24%) 80 (76%)	

Mean (standard deviation) for all participants at each time point. After accounting for missing data and outliers, *N* = 170 included for Time 1 analyses, *N* = 262 included for Time 2 analyses, and *N* = 102 included for longitudinal analyses.

^a^Time 1: 2 missing (*N* = 170); Time 2: 4 missing (*N* = 263); Longitudinal: 2 missing (*N* = 103).

^b^Time 2: 1 missing (*N* = 266); Longitudinal: 1 missing (*N* = 104).

### MRI acquisition and processing

T1-weighted (3D fast spoiled gradient echo, TR = 8.084 ms, TE = 3.164 ms) and diffusion-weighted images (DWIs) (51 diffusion directions, *b* value = 1,000 s/mm^2^, integrated with a pair of *b* = 0 images with opposite phase encode polarity, TR = 9,700 ms, TE = 80–84 ms) were acquired at UCSD on two GE 3.0 Tesla Discovery 750 × scanners (GE Healthcare, Waukesha, WI, USA) with an eight-channel phased array head coil.

### Image preprocessing

Images were preprocessed at the UCSD Center for Multimodal Imaging Genetics [35, 36]. Briefly, T1-weighted images were corrected for gradient nonlinearity distortions [37] and B1 field inhomogeneity [38] and then rigidly resampled and registered to standard space. DWIs were corrected for eddy current distortion [39], head motion [40], B0 distortions [41], and gradient nonlinearity distortions [37]. The *b* = 0 images were registered to T1 images using mutual information [42] and then rigidly resampled into a standard orientation relative to the atlas-registered T1, with 2-mm isotropic resolution. All images were visually inspected to exclude data with severe scanner artifacts or excessive head motion from subsequent analyses.

### Construction of the structural connectome

**Fig. 2:**
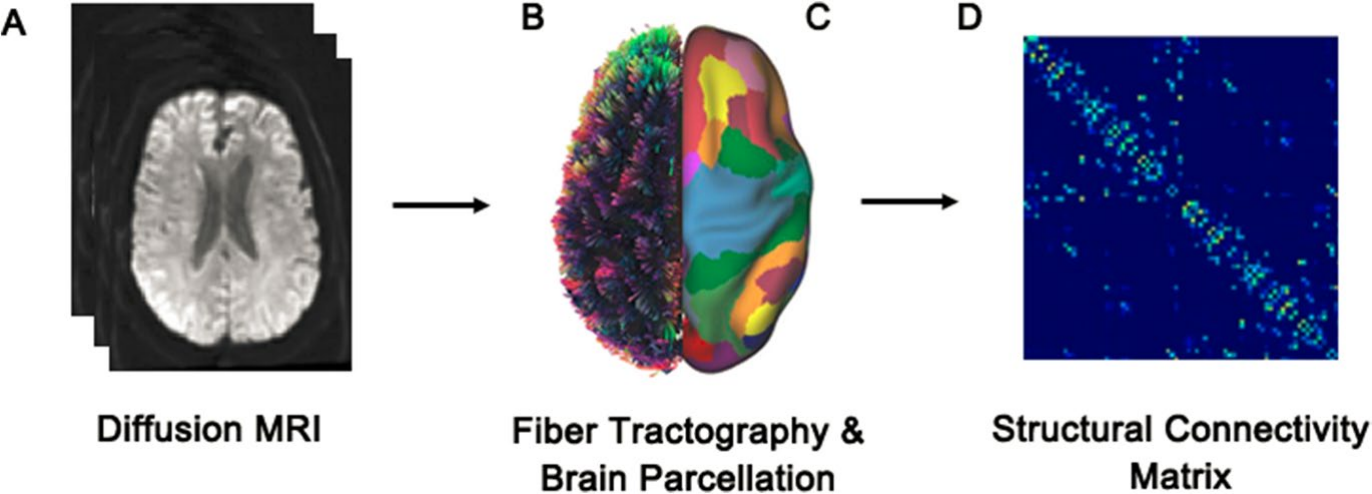
Construction of the structural connectome. **A** Diffusion MRI data were acquired and preprocessed. **B** From these data, white matter streamlines were reconstructed via whole-brain tractography. **C**, **D** Streamlines connecting each pair of brain regions (400 parcels obtained from the Schaefer atlas) were counted to build a structural connectivity matrix, with each cell representing the number of streamlines between two regions

Preprocessed diffusion MRI data (Fig. 2A) was reconstructed in DSI Studio (http://dsi-studio.labsolver.org/) using q-space diffeomorphic reconstruction (QSDR) [43]. Quantitative anisotropy (QA) values were computed in each voxel and subsequently used to warp the brain to a template QA volume in Montreal Neurological Institute (MNI) space using the statistical parametric mapping (SPM) nonlinear registration algorithm. Once in MNI space, spin density functions were reconstructed again with a mean diffusion distance of 1.25 mm using three fiber orientations per voxel. Whole-brain fiber tractography (Fig. 2B) was performed using a deterministic fiber tracking algorithm with an angular cutoff of 35°, step size of 1.0 mm, minimum length of 10 mm, maximum length of 200 mm, and a QA threshold determined by DWI signal in the colony-stimulating factor. Whole-brain fiber tracking was performed until 1,000,000 streamlines were reconstructed. We used the Schaefer atlas [17] that subdivided the brain into 400 cortical parcels (Fig. 2C), yielding a structural connectivity matrix (Fig. 2D) for each participant with brain parcels as nodes and the number of streamlines connecting any pair of parcels as weighted edges [6].

### Computation of network modal controllability

Modal controllability was computed using previously established mathematical formulations [3] and code (https://complexsystemsupenn.com/s/controllability_code-smb8.zip) in MATLAB [3, 8]. Each participant’s structural connectivity matrix was first normalized by dividing each element by the largest absolute eigenvalue of the matrix plus one [44]. The modal controllability measures were then calculated for each parcel from the normalized matrix for each participant. We then calculated the mean modal controllability over all regions in the control network and the multiple demand system. Consequently, there were two modal controllability measures computed for each participant, one summary metric corresponding to each network. A list of brain parcels included in each of the networks can be found in Tab s1 and Tab s2.

### Executive function

As described in the introduction, we included the following six well-established neuropsychological tasks from our clinical neuropsychological task battery to compute the executive function factor score: Stroop [45], trail making test [46], category switching [46], letter-number sequencing [47], reading span [48], and digit span [47] (see Supplementary Methods for task descriptions). These tasks were specifically chosen because they are the most relevant tasks tapping into classic subdomains of executive function that comprise a well-established executive function model [21, 22], and they engage high-level cognitive control processes that are critical for exercising executive function. All tasks were administered in a standardized fashion according to the manuals.

For Stroop, a residualized score for the number of correct words identified during the color-word condition (naming colors of words printed in incongruent colors) was used, adjusting for performance on the word condition (reading color words printed in black ink) and the color condition (naming the color of printed strings of Xs). For trail making test, a residualized score for the time spent on the switching trials was used, adjusting for the time on the single-task trials. For category switching, a residualized score for category switching accuracy (number of correct switches) was used, adjusting for the number of correct responses across the category fluency trials. For letter-number sequencing, the total number of trials passed on the letter-number sequencing subtest was used. For reading span, the total number of correct words recalled across the entire task regardless of order was used. For digit span, the total number of correct trials across both the forward and backward conditions in the digit span subtest was used. The reason for using residualized scores is that these scores remove variance accounted for by more elementary task components in order to isolate the executive component. The factor score then computed from these six tasks, which was shown to be highly heritable and factorially invariant across middle age and early old age in a larger sample of VETSA participants (*N* > 1200) [23]. Practice effects were adjusted for all tasks for returning participants [49, 50]. Higher scores indicate better executive function.

### Young adult general cognitive ability

Participants were on average 20 years of age when they completed the Armed Forces Qualification Test (AFQT). The AFQT is a standardized, validated 100-item multiple-choice paper-and-pencil test of general cognitive ability [51]. It includes 4 components: vocabulary, arithmetic, spatial processing, and knowledge and reasoning about tools. AFQT percentile scores were probit transformed and z-scored for analysis. This test is highly correlated with other tests of general cognitive ability such as the Wechsler Adult Intelligence Scale (*r* = 0.84) [52].

### Physical health status

A modification of the Charlson Comorbidity Index was used [53]. One point was assigned for the presence of each of 15 different chronic medical conditions as described previously [30]. Higher scores indicate poorer health. Individuals with scores greater than 3 were recoded as 3 (Time 1: *N* = 7, original data: median = 1, range = 0–6; Time 2: *N* = 17, original data: median = 1, range = 0–6) to improve the overall data distribution.

### Statistical analyses

All statistical analyses were performed using R version 4.1.2 (R Development Core Team 2022). Linear mixed models were performed using the lme4 package [54]. Summaries of linear mixed models were obtained using the jtools package (https://cran.r-project.org/web/packages/jtools/index.html), and p-values were calculated using Satterthwaite degrees of freedom approximation. Multiple comparison correction was applied using the Benjamini–Hochberg false discovery rate (FDR) control. Spearman’s rank correlations were performed using the *cor ()* function. One outlier in the modal controllability of multiple demand system at Time 2 was removed from analyses, as it was 3 standard deviations below the mean. For all analyses, the inclusion of the outlier did not change our results. The outlier was not excluded for Spearman’s rank correlational analyses as they are less sensitive to outliers.

For cross-sectional analyses at each time point, we built our linear mixed models by setting the executive function factor score as the dependent variable and modal controllability for each network as the independent variable. Age, race/ethnicity, health status, and young adult general cognitive ability were included as covariates in all models. Because there were twin pairs in our sample, we also included twin pair ID as a random intercept in linear mixed models to account for correlated outcomes. For longitudinal analyses, difference in the executive function factor score (Time 1 minus Time 2) was the dependent variable and difference in modal controllability (Time 1 minus Time 2) was the independent variable. Differences in age and health status (Time 1 minus Time 2), race/ethnicity, and general cognitive ability were included as covariates in all models. We confirm that the investigators have access to the data used in all analyses.

## Results

### Topography of modal controllability across Time 1 and Time 2

**Fig. 3:**
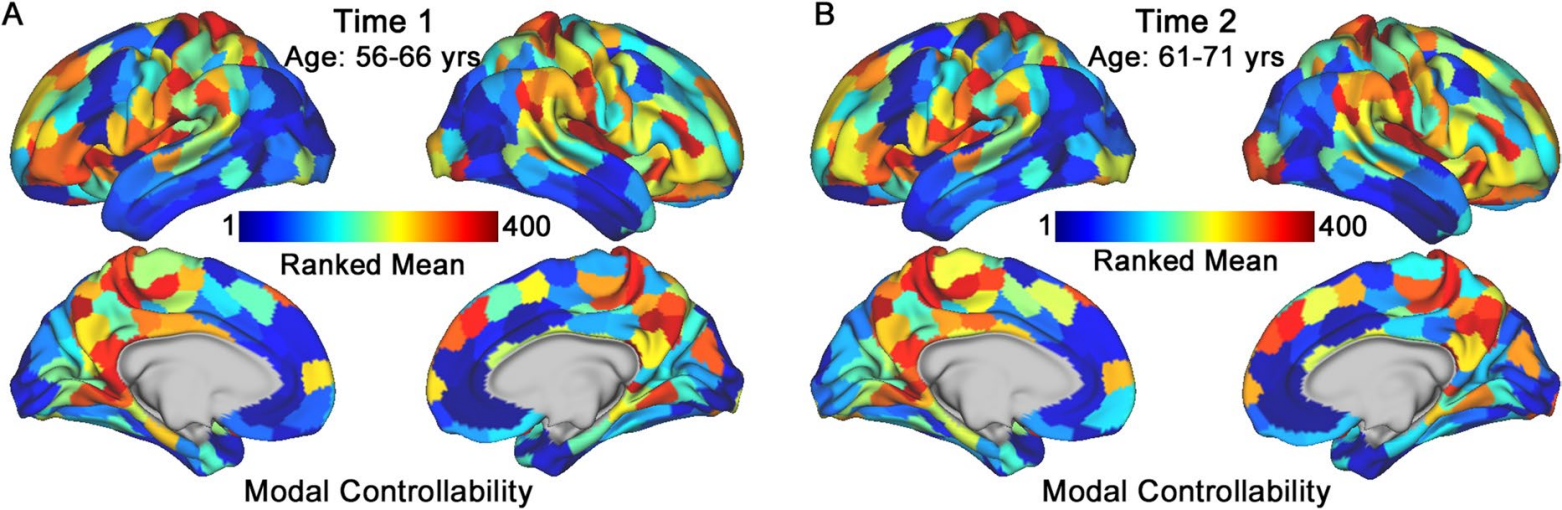
Regional modal controllability at Time 1 and Time 2. Regional modal controllability was computed from all participants at each time point by taking the group average of controllability values at each parcel. Region modal controllability values were then ranked for all 400 cortical parcels at each time point and plotted on a surface visualization. Warmer colors indicate larger values of regional modal controllability

Figure 3 depicts the regional modal controllability of all 400 cortical parcels as ranked means across the brain at each time point. The spatial distributions of modal controllability were highly similar when comparing between the two time points (*ρ* = 0.96, *p* < 2.2 × 10^−16^; Fig. 3A, B), suggesting that the topography of modal controllability (but not necessarily controllability strengths) remained stable over a period of 5–6 years.

### Cross-sectional associations of modal controllability and executive function

**Fig. 4:**
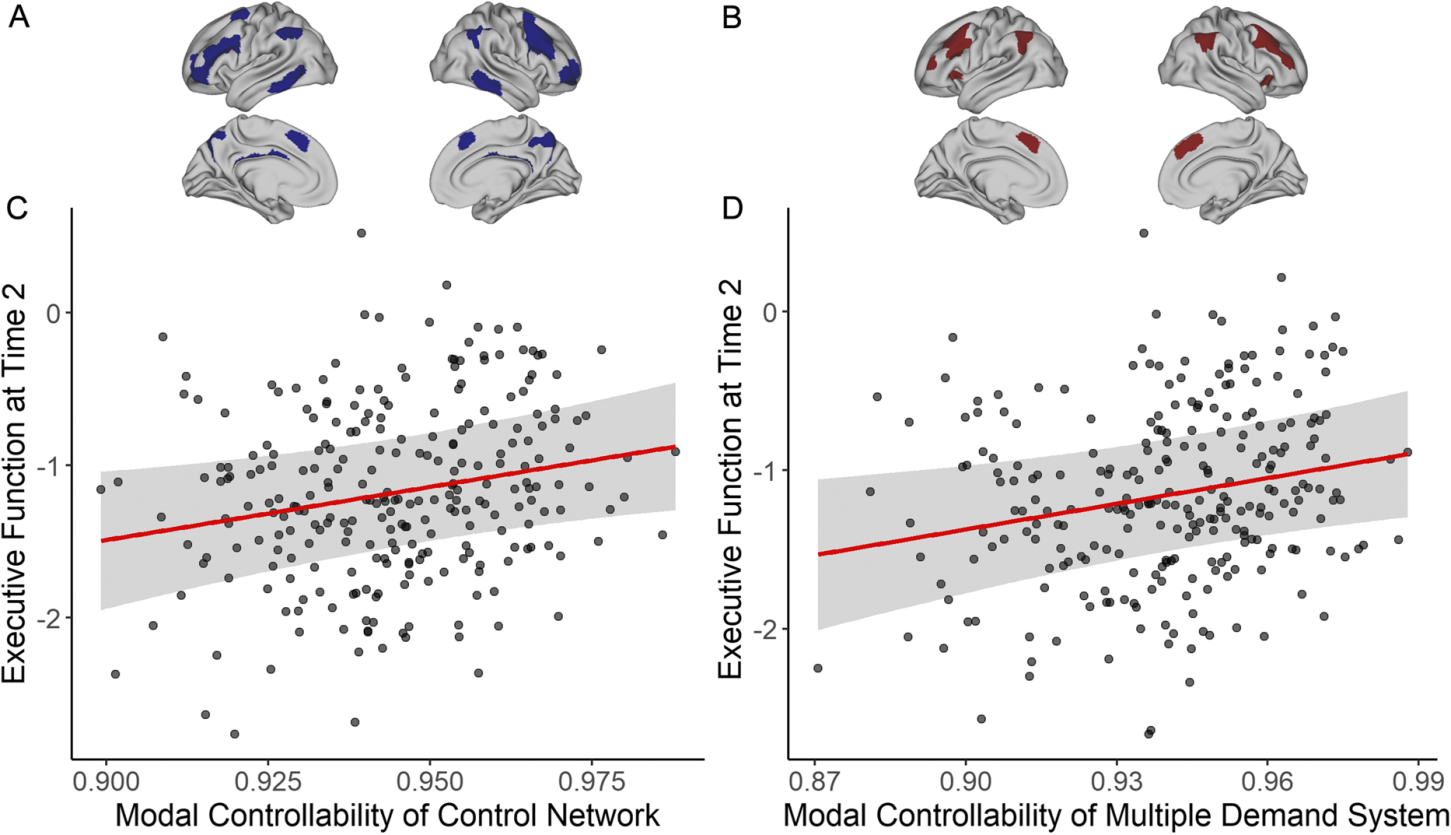
Modal controllability and executive function at Time 2. **A** The control network. **B** The multiple demand system. **C** Main effect of modal controllability of the control network plotted against executive function (adjusted for the effects of covariates) at Time 2. **D** Main effect of modal controllability of the multiple demand system plotted against executive function (adjusted for the effects of covariates) at Time 2

To examine whether modal controllability of the multiple demand system or the control network serves as a predictor of executive function, we estimated network-level modal controllability (i.e., taking the mean of controllability values across all regions within a network) cross-sectionally at each time point (Time 1: *N* = 172; Time 2: *N* = 267). As predicted, we observed a significant main effect at Time 2 showing that higher modal controllability in the control network (Fig. 4A) was associated with better executive function performance (*β* = 0.12, *t* = 2.43, *p* = 0.016, *p*_FDR_ = 0.032, 95% CI [0.02, 0.22]; Fig. 4C). Likewise, modal controllability of the multiple demand system (Fig. 4B) was positively associated with executive function at Time 2 (*β* = 0.12, *t* = 2.50, *p* = 0.013, *p*_FDR_ = 0.032, 95% CI [0.03, 0.22]) (Fig. 4D). Although the associations were in the expected direction, modal controllability of either network was not significantly associated with executive function at Time 1 (control: *β* = 0.09, *t* = 1.46, *p* = 0.143, *p*_FDR_ = 0.143, 95% CI [− 0.03, 0.22]; multiple demand: *β* = 0.10, *t* = 1.59, *p* = 0.113, *p*_FDR_ = 0.143, 95% CI [− 0.02, 0.23]) (Tab s3 and Tab s4).

Both health status and young adult general cognitive ability were significant predictors of executive function at both Time 1 and Time 2 (Tab s3 and Tab s4), consistent with what we found previously in the full VETSA sample [30]. We included these two specific covariates to conduct a more stringent test of whether modal controllability is a predictor that can explain additional variance in executive function in older adults above and beyond the effects of physical health status and earlier general cognitive ability. Collectively, these results showed that individuals whose control network and multiple demand system have a greater ability to make distant or effortful transitions between mental states have better executive function in later life.

### Longitudinal associations of changes in modal controllability and executive function

Following our cross-sectional analyses, we performed longitudinal analyses on modal controllability of the control network and that of the multiple demand network in participants with data at both time points (*N* = 105). We predicted that age-related decline in executive function from Time 1 to Time 2 would correspond to decreases in modal controllability for both networks.

**Fig. 5:**
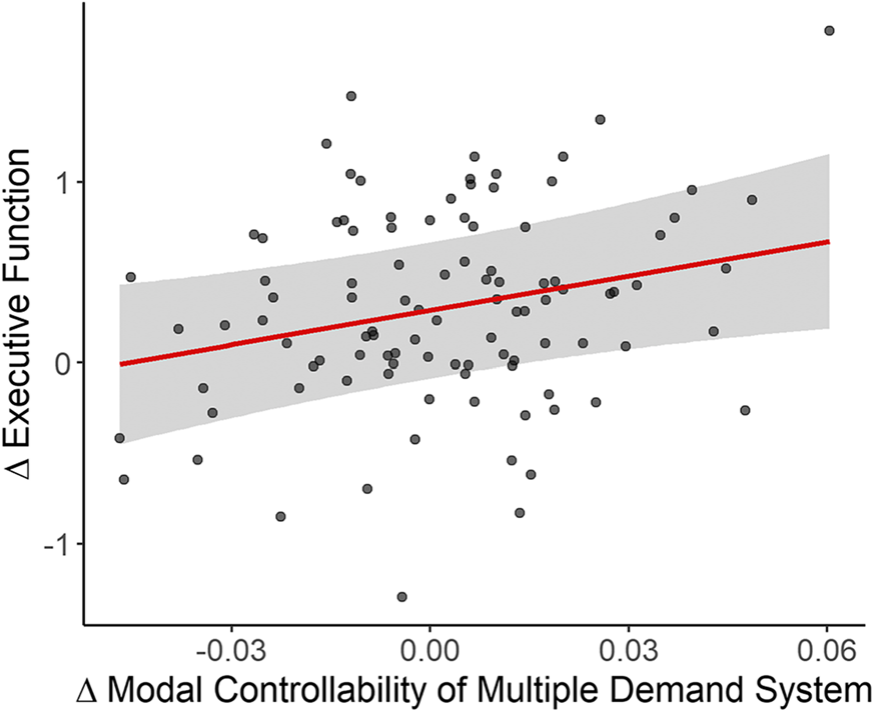
Longitudinal changes in modal controllability of the multiple demand system and executive function. The plot depicts the association of changes (Δ) in modal controllability and executive function (adjusted for all covariates) from Time 1 to Time 2. The positive slope indicates that *greater* reduction of modal controllability is associated with *greater* decline in executive function and vice versa

We detected a significant association such that changes in modal controllability of the multiple demand system were positively related to changes in executive function (*β* = 0.14, *t* = 2.48, *p* = 0.013, 95% CI [0.03, 0.24]) (Fig. 5), after controlling for covariates (Tab s5). Changes in modal controllability of the control network were also positively associated with changes in executive function but that association did not reach the threshold for statistical significance (*β* = 0.11, *t* = 1.99, *p* = 0.050, 95% CI [0.00, 0.22]) (Tab s6). Thus, individuals who had a greater reduction in the multiple demand system’s ability to facilitate effortful transitions between mental states over a period of 5–6 years experienced a greater decline in executive function.

### Interactions of modal controllability with *APOE* genotype and young adult general cognitive ability

Cognitive aging is known to be influenced by a wide array of factors including genetics and the level of early life cognitive ability [30]. In exploratory cross-sectional analyses, we did not observe any significant *APOE* genotype × modal controllability interaction for either network at either time point (Tab s7 and Tab s8). We also did not detect any interaction with young adult general cognitive ability for either network at either time point (Tab s9 and Tab s10). Overall, our findings indicated that the associations between modal controllability of the two networks and executive function in late midlife were not moderated by *APOE* genotype or by young adult general cognitive ability. Finally, it is worth noting that prior work from our group demonstrated that young adult general cognitive ability accounts for roughly 10% of variance in late midlife executive function [30]. However, while young adult general cognitive ability was significantly correlated with executive function at both time points (Time 1: *r*(170) = 0.36, *p* < 0.0001; Time 2: *r*(262) = 0.45, *p* < 0.0001), it was not correlated with modal controllability of either network at either time point (Tab s11 and Tab s12).

## Discussion

Declines in executive function are a hallmark of cognitive aging and aging-related disorders, but the neural mechanisms underlying these cognitive declines have not been fully elucidated. We provided a mechanistic explanation for aging-related changes in executive function based on understanding how the structural connectome supports brain state transitions to enable these effortful cognitive processes [3, 4]. We showed that changes in executive function can be explained by changes in the ability of the multiple demand system to support the brain’s transition to difficult-to-reach states that are cognitively effortful and demanding. Taken together, our findings suggest that a putative neural mechanism of cognitive aging may involve decreased modal controllability in structural brain networks.

A prominent theoretical framework provides strong evidence for executive functions being both domain-general (i.e., tapping into a common core capacity) and domain-specific (i.e., separable by tasks) [21, 22]. This conceptual framework—the unity and diversity model—is in line with most studies on cognitive control that show domain-general task activations distributed across the brain, along with task-specific neural activations [13, 18]. In the present study, we tested our hypotheses in two somewhat overlapping but different networks that have been linked to these types of cognitive functions. As discussed previously, the multiple demand system is a domain-general cognitive core encompassing a set of key regions from frontoparietal control and cingulo-opercular networks [16] that are consistently activated in a wide range of cognitively demanding task conditions (i.e., high working memory load > low working memory load) across various cognitive domains [12, 15]. On the other hand, the control network consists of frontal and parietal regions (including frontoparietal regions within the multiple demand system) broadly relevant for cognitive and control processes [17]. The control network does not include regions from the salience/cingulo-opercular network implicated in response inhibition and maintenance of tonic alertness [20]. Although modal controllability of both networks exhibited the same magnitude of association with executive function cross-sectionally, the relationship appeared to be somewhat stronger in the multiple demand system than the control network when examined longitudinally.

One tenable explanation is that the executive function factor used in our study—referred to as Common Executive Function in the unity and diversity model—represents common shared variance across multiple executive function tasks that captures domain-general executive function capacity. Therefore, change in executive function was preferentially predicted by change in modal controllability of the multiple demand system, a network that is not only domain-general, but also critical for supporting effortful cognitive control processes engaged by executive function tasks [15], which tend to become increasingly more cognitively effortful and demanding as individuals advanced in age. In particular, a number of studies have shown that cognitive effort is experienced as subjectively more costly in older adults than in young adults when matched on task conditions and difficulty [55–57]. Thus, the longitudinal association with multiple demand system may also reflect the fact that older adults experienced these executive function tasks as overall cognitively demanding and effortful. Relatedly, the barely significant longitudinal association of the control network is likely due to this very reason, such that the control network regions are not specific to effortful cognitive control processes. Thus, it is likely not capturing aging-related increases in cognitive effort and subjective perceptions of effort for the same set of executive function tasks across two time points. Nonetheless, future studies may want to both replicate the described findings and examine them with respect to objective and subjective cognitive effort. Additionally, future studies may want to explore potential structural networks relevant for specific subdomains of executive functions (e.g., inhibition) with multiple tasks tapping into a single subdomain.

Cross-sectional associations between modal controllability and executive function were not observed at Time 1 but were detected at Time 2. Given that the significant associations at Time 2 were fairly modest (*β* = 0.12), one possible explanation for the lack of associations at Time 1 is likely to be the smaller sample size (*N* ≈ 170) relative to Time 2 (*N* ≈ 260), which may not be sufficiently powered to detect these modest brain-behavior relationships. Indeed, the effect sizes for both networks at each time point were small and virtually the same across time (multiple demand: partial eta-squared = 0.02 at both time points; control network: partial eta-squared = 0.01 at Time 1, and 0.02 at Time 2). In light of prior work on controllability [8, 9], these modest associations are in fact expected.

Finally, modal controllability still explained some variance in executive function above and beyond that explained by physical health [30] and young adult general cognitive ability, an index of cognitive reserve [27]. We define cognitive reserve as a person’s overall cognitive resources at a given point in time [27]. The use of young adult cognitive reserve ensures that the reserve index is unaffected by aging-related cognitive declines. By including these two variables as covariates, we undoubtedly attenuated the amount of variance explained by modal controllability. Nonetheless, our longitudinal results provide stronger support for the cognitive relevance of modal controllability, as changes in executive function across the two assessments were only predicted by changes in modal controllability, but not by young adult cognitive reserve (general cognitive ability) or physical health. This suggests that earlier cognitive ability and late life physical health may be good predictors of late life executive function, but may not be good predictors of aging-related executive function declines. Put another way, physical health and young adult cognitive reserve may predict the intercept for executive function later in life, but modal controllability may predict its slope.

Our results have important potential clinical implications for the development of intervention approaches to ameliorate aging-related cognitive declines. For instance, one recent study in healthy young adults combined functional MRI and DTI and found that modal controllability of dorsolateral prefrontal cortex, a key region in the control network and the multiple demand system, predicted functional activations during a working memory task, particularly in a high working memory load condition. Importantly, applying repetitive transcranial magnetic stimulation (rTMS) to the same regions of the dorsolateral prefrontal cortex resulted in behavioral improvement in task performance [58]. This study demonstrated that intervention approaches targeting critical brain modal control hubs (i.e., regions with high modal controllability) could substantially improve their efficacy in bolstering cognitive performance. Therefore, it may be possible to target aging-related executive function declines in older adults by stimulating regions within the multiple demand system through different intervention approaches.

Finally, several limitations should be noted. The all-male, mostly white non-Hispanic sample of our study presents challenges to generalizability. Based on a prior study that showed sex differences in network controllability in relation to executive functions in youths ages 7 to 22 [10], it will be important to directly investigate where there could be sex differences in the cross-sectional and longitudinal associations between modal controllability and executive function in older adults. Of note, our narrow age range at each assessment is a strength because it allows for investigation of longitudinal within-person change without potential confounding by large baseline age differences. It thus provided a window into change in early old age, but future work should examine changes in controllability and executive function across a broad age range in later life.

In conclusion, executive functions are fundamental to successful daily functioning and particularly for healthy aging. Addressing the neural mechanisms underpinning executive function declines in older adults has critical empirical and clinical implications for cognitive aging and related disorders. Our results suggest that changes in structural network controllability are linked to declines in executive function in older adults. These findings open up new avenues for basic research examining relationships between individual differences in structural network controllability and other domains of cognition. Moreover, the findings provide network control hubs that could be directly targeted by clinical approaches to potentially ameliorate executive dysfunction in vulnerable populations.

## Supplementary Information

Below is the link to the electronic supplementary material.

Supplementary file1 (DOCX 70 KB)

## References

[1] Diamond A (2013). Executive functions. Annu Rev Psychol.

[2] Hofmann W, Schmeichel BJ, Baddeley AD (2012). Executive functions and self-regulation. Trends Cogn Sci.

[3] Gu S, Pasqualetti F, Cieslak M, Telesford QK, Yu AB, Kahn AE (2015). Controllability of structural brain networks. Nat Commun.

[4] Hermundstad AM, Brown KS, Bassett DS, Aminoff EM, Frithsen A, Johnson A (2014). Structurally-constrained relationships between cognitive states in the human brain. PLoS Comput Biol.

[5] Yu M, Sporns O, Saykin AJ (2021). The human connectome in Alzheimer disease - relationship to biomarkers and genetics. Nat Rev Neurol.

[6] Bassett DS, Zurn P, Gold JI (2018). On the nature and use of models in network neuroscience. Nat Rev Neurosci.

[7] Senden M, Deco G, de Reus MA, Goebel R, van den Heuvel MP (2014). Rich club organization supports a diverse set of functional network configurations. Neuroimage.

[8] Tang E, Giusti C, Baum GL, Gu S, Pollock E, Kahn AE (2017). Developmental increases in white matter network controllability support a growing diversity of brain dynamics. Nat Commun.

[9] Lee WH, Rodrigue A, Glahn DC, Bassett DS, Frangou S (2020). Heritability and cognitive relevance of structural brain controllability. Cereb Cortex.

[10] Cornblath EJ, Tang E, Baum GL, Moore TM, Adebimpe A, Roalf DR (2019). Sex differences in network controllability as a predictor of executive function in youth. Neuroimage.

[11] Betzel RF, Gu S, Medaglia JD, Pasqualetti F, Bassett DS (2016). Optimally controlling the human connectome: the role of network topology. Sci Rep.

[12] Fedorenko E, Duncan J, Kanwisher N (2013). Broad domain generality in focal regions of frontal and parietal cortex. Proc Natl Acad Sci U S A.

[13] Braver TS, Kizhner A, Tang R, Freund MC, Etzel JA. The dual mechanisms of cognitive control project. J Cogn Neurosci. 2021:1–26 10.1162/jocn_a_01768 PMC10069323 34407191

[14] Tang R, Etzel JA, Kizhner A, Braver TS (2021). Frontoparietal pattern similarity analyses of cognitive control in monozygotic twins. Neuroimage.

[15] Assem M, Glasser MF, Van Essen DC, Duncan J (2020). A domain-general cognitive core defined in multimodally parcellated human cortex. Cereb Cortex.

[16] Duncan J (2010). The multiple-demand (MD) system of the primate brain: mental programs for intelligent behaviour. Trends Cogn Sci.

[17] Schaefer A, Kong R, Gordon EM, Laumann TO, Zuo XN, Holmes AJ (2018). Local-global parcellation of the human cerebral cortex from intrinsic functional connectivity MRI. Cereb Cortex.

[18] Cole MW, Reynolds JR, Power JD, Repovs G, Anticevic A, Braver TS (2013). Multi-task connectivity reveals flexible hubs for adaptive task control. Nat Neurosci.

[19] Niendam TA, Laird AR, Ray KL, Dean YM, Glahn DC, Carter CS (2012). Meta-analytic evidence for a superordinate cognitive control network subserving diverse executive functions. Cogn Affect Behav Neurosci.

[20] Menon V, D'Esposito M (2022). The role of PFC networks in cognitive control and executive function. Neuropsychopharmacology.

[21] Miyake A, Friedman NP (2012). The nature and organization of individual differences in executive functions: four general conclusions. Curr Dir Psychol Sci.

[22] Friedman NP, Miyake A (2017). Unity and diversity of executive functions: individual differences as a window on cognitive structure. Cortex.

[23] Gustavson DE, Panizzon MS, Elman JA, Franz CE, Reynolds CA, Jacobson KC (2018). Stability of genetic and environmental influences on executive functions in midlife. Psychol Aging.

[24] Gustavson DE, Panizzon MS, Franz CE, Friedman NP, Reynolds CA, Jacobson KC (2018). Genetic and environmental architecture of executive functions in midlife. Neuropsychology.

[25] Friedman NP, Robbins TW (2022). The role of prefrontal cortex in cognitive control and executive function. Neuropsychopharmacology.

[26] Kunkle BW, Grenier-Boley B, Sims R, Bis JC, Damotte V, Naj AC (2019). Genetic meta-analysis of diagnosed Alzheimer’s disease identifies new risk loci and implicates Aβ, tau, immunity and lipid processing. Nat Genet.

[27] Kremen WS, Elman JA, Panizzon MS, Eglit GML, Sanderson-Cimino M, Williams ME, et al. Cognitive reserve and related constructs: a unified framework across cognitive and brain dimensions of aging. Front Aging Neurosci. 2022;14 10.3389/fnagi.2022.834765 PMC9196190 35711905

[28] Franz CE, Hatton SN, Elman JA, Warren T, Gillespie NA, Whitsel NA (2021). Lifestyle and the aging brain: interactive effects of modifiable lifestyle behaviors and cognitive ability in men from midlife to old age. Neurobiol Aging.

[29] Eglit GML, Elman JA, Panizzon MS, Sanderson-Cimino M, Williams ME, Dale AM, et al. Paradoxical cognitive trajectories in men from earlier to later adulthood. Neurobiol Aging. 2021. 10.1016/j.neurobiolaging.2021.10.002 PMC8715388 34785406

[30] Kremen WS, Beck A, Elman JA, Gustavson DE, Reynolds CA, Tu XM (2019). Influence of young adult cognitive ability and additional education on later-life cognition. Proc Natl Acad Sci U S A.

[31] Kremen WS, Thompson-Brenner H, Leung YM, Grant MD, Franz CE, Eisen SA (2006). Genes, environment, and time: The Vietnam Era Twin Study of Aging (VETSA). Twin Res Hum Genet.

[32] Kremen WS, Franz CE, Lyons MJ (2013). VETSA: The Vietnam Era Twin Study of Aging. Twin Res Hum Genet.

[33] Kremen WS, Franz CE, Lyons MJ (2019). Current status of the Vietnam Era Twin Study of Aging (VETSA). Twin Res Hum Genet.

[34] Schoenborn CA, Heyman KM (2009). Health characteristics of adults aged 55 years and over: United States, 2004–2007. Natl Health Stat Report.

[35] McEvoy LK, Fennema-Notestine C, Eyler LT, Franz CE, Hagler DJ, Lyons MJ (2015). Hypertension-related alterations in white matter microstructure detectable in middle age. Hypertension.

[36] Vuoksimaa E, Panizzon MS, Hagler DJ, Hatton SN, Fennema-Notestine C, Rinker D (2017). Heritability of white matter microstructure in late middle age: a twin study of tract-based fractional anisotropy and absolute diffusivity indices. Hum Brain Mapp.

[37] Jovicich J, Czanner S, Greve D, Haley E, van der Kouwe A, Gollub R (2006). Reliability in multi-site structural MRI studies: effects of gradient non-linearity correction on phantom and human data. Neuroimage.

[38] Sled JG, Zijdenbos AP, Evans AC (1998). A nonparametric method for automatic correction of intensity nonuniformity in MRI data. IEEE Trans Med Imaging.

[39] Zhuang J, Hrabe J, Kangarlu A, Xu D, Bansal R, Branch CA (2006). Correction of eddy-current distortions in diffusion tensor images using the known directions and strengths of diffusion gradients. J Magn Reson Imaging.

[40] Hagler DJ, Ahmadi ME, Kuperman J, Holland D, McDonald CR, Halgren E (2009). Automated white-matter tractography using a probabilistic diffusion tensor atlas: application to temporal lobe epilepsy. Hum Brain Mapp.

[41] Holland D, Kuperman JM, Dale AM (2010). Efficient correction of inhomogeneous static magnetic field-induced distortion in Echo Planar Imaging. Neuroimage.

[42] Wells WM, Viola P, Atsumi H, Nakajima S, Kikinis R (1996). Multi-modal volume registration by maximization of mutual information. Med Image Anal.

[43] Yeh FC, Wedeen VJ, Tseng WY (2011). Estimation of fiber orientation and spin density distribution by diffusion deconvolution. Neuroimage.

[44] Karrer TM, Kim JZ, Stiso J, Kahn AE, Pasqualetti F, Habel U (2020). A practical guide to methodological considerations in the controllability of structural brain networks. J Neural Eng.

[45] Golden CJ. Stroop color and word test. Multi-Health Systems; 2003.

[46] Delis DC, Kaplan E, Kramer JH (2001). Delis-Kaplan Executive Function System (D-KEFS).

[47] Wechsler D (1997). Wechsler Memory Scale (WMS-III).

[48] Daneman M, Merikle PM (1980). Working memory and language comprehension: a meta-analysis. Psychon Bull.

[49] Elman JA, Jak AJ, Panizzon MS, Tu XM, Chen T, Reynolds CA (2018). Underdiagnosis of mild cognitive impairment: a consequence of ignoring practice effects. Alzheimers Dement (Amst).

[50] Sanderson-Cimino M, Elman JA, Tu XM, Gross AL, Panizzon MS, Gustavson DE (2022). Cognitive practice effects delay diagnosis of MCI: implications for clinical trials. Alzheimers Dement (N Y).

[51] Uhlaner JE, Bolanovich DJ. Development of the Armed Forces Qualification Test and predecessor army screening tests, 1946–1950. Pay Research Bureau (PRB) Report (1952), Article AD0000191. 1952.

[52] Lyons MJ, York TP, Franz CE, Grant MD, Eaves LJ, Jacobson KC (2009). Genes determine stability and the environment determines change in cognitive ability during 35 years of adulthood. Psychol Sci.

[53] Charlson M, Szatrowski TP, Peterson J, Gold J (1994). Validation of a combined comorbidity index. J Clin Epidemiol.

[54] Bates D, Mächler M, Bolker B, Walker S (2015). Fitting linear mixed-effects models using lme4. J Stat Softw.

[55] Hess TM, Ennis GE (2012). Age differences in the effort and costs associated with cognitive activity. J Gerontol B Psychol Sci Soc Sci.

[56] Westbrook A, Kester D, Braver TS (2013). What is the subjective cost of cognitive effort? Load, trait, and aging effects revealed by economic preference. PLoS ONE.

[57] Ennis GE, Hess TM, Smith BT (2013). The impact of age and motivation on cognitive effort: implications for cognitive engagement in older adulthood. Psychol Aging.

[58] Beynel L, Deng L, Crowell CA, Dannhauer M, Palmer H, Hilbig S (2020). Structural controllability predicts functional patterns and brain stimulation benefits associated with working memory. J Neurosci.

