# Supplementary material for: Longitudinal association of executive function and structural network controllability in the aging brain
Source: GeroScience. 2022 Oct 21;45(2):837–49. doi: 10.1007/s11357-022-00676-3 (PMC9886719; doi:10.1007/s11357-022-00676-3)
Supplement: Supplementary file 1 — Supplementary file1 (DOCX 70 KB) [file 11357_2022_676_MOESM1_ESM.docx]

**Longitudinal Changes in Executive Function and Structural Network Controllability in the Aging Brain**

**Supplementary Materials**

**Supplemental Methods**

Quality Control for Fiber Tractography

**Tab s1**. Multiple Demand System: Center of mass and corresponding Schaefer 400, 17 networks label for each parcellation region

**Tab s2**. Control Network: Center of mass and corresponding Schaefer 400, 17 networks label for each parcellation region

Neuropsychological Tasks of Executive Function and Factor Score Computation

**Supplemental Results**

**Tab s3**. Control Network Modal Controllability: Linear mixed effects models predicting executive functions cross-sectionally at Time 1 and Time 2

**Tab s4**. Multiple Demand System Modal Controllability: Linear mixed effects models predicting executive functions cross-sectionally at Time 1 and Time 2

**Tab s5**. Multiple Demand Network Modal Controllability: Linear mixed effects models predicting changes in executive functions longitudinally from Time 1 to Time 2

**Tab s6**. Control Network Modal Controllability: Linear mixed effects models predicting changes in executive functions longitudinally from Time 1 to Time 2

**Tab s7.** Control Network Modal Controllability X APOE genotype: Linear mixed effects models predicting executive functions cross-sectionally at Time 1 and Time 2

**Tab s8.** Multiple Demand System Modal Controllability X APOE genotype: Linear mixed effects models predicting executive functions cross-sectionally at Time 1 and Time 2

**Tab s9.** Control Network Modal Controllability X Young Adult General Cognitive Ability: Linear mixed effects models predicting executive functions cross-sectionally at Time 1 and Time 2

**Tab s10.** Multiple Demand System Modal Controllability X Young Adult General Cognitive Ability: Linear mixed effects models predicting executive functions cross-sectionally at Time 1 and Time 2

**Tab s11.** Correlations among Variables at Time 1 in Cross-sectional Participants

**Tab s12.** Correlations among Variables at Time 2 in Cross-sectional Participants

# **Supplementary Methods**

## Quality Control for Fiber Tractography

Before the main analyses, each participant’s whole-brain fiber tractography were examined visually for false tracks or any abnormalities (e.g., wrong fiber directions). We rated each participant’s fiber tractography result (0=no or minimal number of false tracks, 1=some false tracks but in localized areas, covering less than 10% of the brain, 2=widespread false tracks in multiple areas (greater than 2), covering more than 10% of the brain, or wrong fiber directions). Participants who scored a 2 were excluded from further analyses. Additionally, we compared modal controllability values between group that scored 0 (Time 1: N=104; Time 2: N=92) and group that scored 1 (Time 1: N=68; Time 2: N=175), and confirmed that the two groups did not differ significantly in controllability values at either time point (p>0.1).

## Neuropsychological Tasks of Executive Function and Factor Score Computation

Common executive function factor was calculated based on six neuropsychological tasks of executive function. All tasks were administered in a standardized fashion based on the manuals. In *Stroop* [1], participants were asked to read color words printed in black ink (word condition), name the color of printed strings of Xs (color condition), and the colors of words printed in incongruent colors (color-word condition). In *Trails Making Test* [2], participants were asked to circles with numbers and circles with letters on the page, and must connect them in sequence while alternating between number and letter. There were condition 2 (number sequencing), condition 3 (letter sequencing), and condition 4 (letter-number switching). In *Category Switching* [2], there were two category fluency trials (think of animals and boys’ names), and the category switching condition (alternate between naming fruits and furniture). The *Letter-Number Sequencing Task* [3] involves re-sequence letter-number strings of increasing size with the numbers in ascending order, followed by the letters in alphabetical order. In *Reading Span* [4], participants were asked to read multiple sentences aloud, presented one at a time on a computer screen. After reading sentences aloud, participants were instructed to recall the last word of each sentence in order. All participants completed 5 trials each of length 2, 3, and 4 sentences. In *Digit Span* [3], participants were asked to repeat a list of single digit number out loud in the same order as the examiner (forward condition) and in the reverse order as the examiner (backward condition). A confirmatory factor analysis was then performed based on these scores from all six tasks to derive a common executive function factor score for each participant.

# **Tab s1. Multiple Demand System: Center of mass and corresponding Schaefer 400, 17 networks label for each parcellation region**

| Parcel Number | Hemisphere | Network | ROI Name | X | Y | Z |
| --- | --- | --- | --- | --- | --- | --- |
| 101 | Left | Salience/Ventral Attention | 17Networks_LH_SalVentAttnB_PFCl_1 | -38 | 50 | 10 |
| 103 | Left | Salience/Ventral Attention | 17Networks_LH_SalVentAttnB_PFCl_3 | -36 | 32 | 38 |
| 105 | Left | Salience/Ventral Attention | 17Networks_LH_SalVentAttnB_Ins_2 | -32 | 24 | 0 |
| 122 | Left | Control | 17Networks_LH_ContA_IPS_1 | -28 | -74 | 42 |
| 124 | Left | Control | 17Networks_LH_ContA_IPS_3 | -34 | -62 | 48 |
| 125 | Left | Control | 17Networks_LH_ContA_IPS_4 | -44 | -42 | 46 |
| 131 | Left | Control | 17Networks_LH_ContA_PFCl_2 | -44 | 20 | 26 |
| 132 | Left | Control | 17Networks_LH_ContA_PFCl_3 | -40 | 8 | 34 |
| 136 | Left | Control | 17Networks_LH_ContB_IPL_1 | -48 | -60 | 46 |
| 137 | Left | Control | 17Networks_LH_ContB_IPL_2 | -54 | -50 | 44 |
| 138 | Left | Control | 17Networks_LH_ContB_IPL_3 | -42 | -52 | 48 |
| 139 | Left | Control | 17Networks_LH_ContB_PFCd_1 | -30 | 14 | 58 |
| 143 | Left | Control | 17Networks_LH_ContB_PFCmp_1 | -4 | 28 | 48 |
| 174 | Left | Default | 17Networks_LH_DefaultB_IPL_2 | -56 | -54 | 30 |
| 181 | Left | Default | 17Networks_LH_DefaultB_PFCl_1 | -40 | 20 | 48 |
| 304 | Right | Salience/Ventral Attention | 17Networks_RH_SalVentAttnB_IPL_1 | 62 | -38 | 36 |
| 306 | Right | Salience/Ventral Attention | 17Networks_RH_SalVentAttnB_PFCl_1 | 42 | 46 | 14 |
| 309 | Right | Salience/Ventral Attention | 17Networks_RH_SalVentAttnB_Ins_1 | 34 | 22 | -8 |
| 325 | Right | Control | 17Networks_RH_ContA_IPS_1 | 36 | -70 | 46 |
| 326 | Right | Control | 17Networks_RH_ContA_IPS_2 | 54 | -32 | 52 |
| 327 | Right | Control | 17Networks_RH_ContA_IPS_3 | 46 | -44 | 46 |
| 332 | Right | Control | 17Networks_RH_ContA_PFCl_3 | 48 | 28 | 28 |
| 334 | Right | Control | 17Networks_RH_ContA_PFCl_5 | 38 | 10 | 34 |
| 338 | Right | Control | 17Networks_RH_ContB_IPL_1 | 56 | -46 | 32 |
| 339 | Right | Control | 17Networks_RH_ContB_IPL_2 | 54 | -52 | 44 |
| 340 | Right | Control | 17Networks_RH_ContB_IPL_3 | 56 | -40 | 48 |
| 341 | Right | Control | 17Networks_RH_ContB_IPL_4 | 42 | -56 | 48 |
| 342 | Right | Control | 17Networks_RH_ContB_PFCld_1 | 38 | 34 | 38 |
| 343 | Right | Control | 17Networks_RH_ContB_PFCld_2 | 44 | 18 | 44 |
| 345 | Right | Control | 17Networks_RH_ContB_PFCld_4 | 34 | 16 | 56 |
| 350 | Right | Control | 17Networks_RH_ContB_PFCmp_1 | 4 | 28 | 48 |
| 379 | Right | Default | 17Networks_RH_DefaultB_PFCd_3 | 4 | 44 | 40 |

**Note about Multiple Demand System**

A total of 32 parcels from the Schaefer parcellation approximating the core multiple demand system[5] were used to generate the multiple demand system used in this study. Because the core multiple demand system regions were defined in a different parcellation scheme (i.e., Multimodal Parcellation), an iterative approximation procedure was employed, which used the Dice coefficient to quantify how well two collections of vertices from both parcellation schemes match. Detailed steps and code for generating this parcel set can be found at the following link (http://mvpa.blogspot.com/2021/09/approximately-matching-different.html).

# **Tab s2. Control Network: Center of mass and corresponding Schaefer 400, 17 networks label for each parcellation region**

| Parcel Number | Hemisphere | Network | ROI Name | X | Y | Z |
| --- | --- | --- | --- | --- | --- | --- |
| 121 | Left | Control | 17Networks_LH_ContA_Temp_1 | -56 | -62 | 0 |
| 122 | Left | Control | 17Networks_LH_ContA_IPS_1 | -28 | -74 | 42 |
| 123 | Left | Control | 17Networks_LH_ContA_IPS_2 | -58 | -42 | 46 |
| 124 | Left | Control | 17Networks_LH_ContA_IPS_3 | -34 | -62 | 48 |
| 125 | Left | Control | 17Networks_LH_ContA_IPS_4 | -44 | -42 | 46 |
| 126 | Left | Control | 17Networks_LH_ContA_IPS_5 | -34 | -46 | 40 |
| 127 | Left | Control | 17Networks_LH_ContA_PFCd_1 | -22 | 6 | 64 |
| 128 | Left | Control | 17Networks_LH_ContA_PFClv_1 | -48 | 36 | 10 |
| 129 | Left | Control | 17Networks_LH_ContA_PFClv_2 | -42 | 38 | 22 |
| 130 | Left | Control | 17Networks_LH_ContA_PFCl_1 | -50 | 6 | 26 |
| 131 | Left | Control | 17Networks_LH_ContA_PFCl_2 | -44 | 20 | 26 |
| 132 | Left | Control | 17Networks_LH_ContA_PFCl_3 | -40 | 8 | 34 |
| 133 | Left | Control | 17Networks_LH_ContA_Cingm_1 | -4 | 6 | 28 |
| 134 | Left | Control | 17Networks_LH_ContB_Temp_1 | -60 | -36 | -18 |
| 135 | Left | Control | 17Networks_LH_ContB_Temp_2 | -60 | -48 | -10 |
| 136 | Left | Control | 17Networks_LH_ContB_IPL_1 | -48 | -60 | 46 |
| 137 | Left | Control | 17Networks_LH_ContB_IPL_2 | -54 | -50 | 44 |
| 138 | Left | Control | 17Networks_LH_ContB_IPL_3 | -42 | -52 | 48 |
| 139 | Left | Control | 17Networks_LH_ContB_PFCd_1 | -30 | 14 | 58 |
| 140 | Left | Control | 17Networks_LH_ContB_PFClv_1 | -42 | 48 | -6 |
| 141 | Left | Control | 17Networks_LH_ContB_PFClv_2 | -28 | 58 | 0 |
| 142 | Left | Control | 17Networks_LH_ContB_PFClv_3 | -28 | 56 | 12 |
| 143 | Left | Control | 17Networks_LH_ContB_PFCmp_1 | -4 | 28 | 48 |
| 144 | Left | Control | 17Networks_LH_ContC_pCun_1 | -10 | -70 | 32 |
| 145 | Left | Control | 17Networks_LH_ContC_pCun_2 | -10 | -78 | 46 |
| 146 | Left | Control | 17Networks_LH_ContC_pCun_3 | -4 | -64 | 52 |
| 147 | Left | Control | 17Networks_LH_ContC_Cingp_1 | -6 | -40 | 24 |
| 148 | Left | Control | 17Networks_LH_ContC_Cingp_2 | -4 | -22 | 30 |
| 325 | Right | Control | 17Networks_RH_ContA_IPS_1 | 36 | -70 | 46 |
| 326 | Right | Control | 17Networks_RH_ContA_IPS_2 | 54 | -32 | 52 |
| 327 | Right | Control | 17Networks_RH_ContA_IPS_3 | 46 | -44 | 46 |
| 328 | Right | Control | 17Networks_RH_ContA_IPS_4 | 36 | -44 | 44 |
| 329 | Right | Control | 17Networks_RH_ContA_PFCd_1 | 24 | 10 | 58 |
| 330 | Right | Control | 17Networks_RH_ContA_PFCl_1 | 50 | 30 | 18 |
| 331 | Right | Control | 17Networks_RH_ContA_PFCl_2 | 48 | 18 | 24 |
| 332 | Right | Control | 17Networks_RH_ContA_PFCl_3 | 48 | 28 | 28 |
| 333 | Right | Control | 17Networks_RH_ContA_PFCl_4 | 48 | 8 | 24 |
| 334 | Right | Control | 17Networks_RH_ContA_PFCl_5 | 38 | 10 | 34 |
| 335 | Right | Control | 17Networks_RH_ContA_Cingm_1 | 4 | 2 | 30 |
| 336 | Right | Control | 17Networks_RH_ContB_Temp_1 | 62 | -28 | -20 |
| 337 | Right | Control | 17Networks_RH_ContB_Temp_2 | 64 | -42 | -10 |
| 338 | Right | Control | 17Networks_RH_ContB_IPL_1 | 56 | -46 | 32 |
| 339 | Right | Control | 17Networks_RH_ContB_IPL_2 | 54 | -52 | 44 |
| 340 | Right | Control | 17Networks_RH_ContB_IPL_3 | 56 | -40 | 48 |
| 341 | Right | Control | 17Networks_RH_ContB_IPL_4 | 42 | -56 | 48 |
| 342 | Right | Control | 17Networks_RH_ContB_PFCld_1 | 38 | 34 | 38 |
| 343 | Right | Control | 17Networks_RH_ContB_PFCld_2 | 44 | 18 | 44 |
| 344 | Right | Control | 17Networks_RH_ContB_PFCld_3 | 42 | 6 | 50 |
| 345 | Right | Control | 17Networks_RH_ContB_PFCld_4 | 34 | 16 | 56 |
| 346 | Right | Control | 17Networks_RH_ContB_PFClv_1 | 36 | 38 | -14 |
| 347 | Right | Control | 17Networks_RH_ContB_PFClv_2 | 28 | 54 | -14 |
| 348 | Right | Control | 17Networks_RH_ContB_PFClv_3 | 42 | 50 | -6 |
| 349 | Right | Control | 17Networks_RH_ContB_PFClv_4 | 28 | 60 | 2 |
| 350 | Right | Control | 17Networks_RH_ContB_PFCmp_1 | 4 | 28 | 48 |
| 351 | Right | Control | 17Networks_RH_ContC_pCun_1 | 16 | -64 | 28 |
| 352 | Right | Control | 17Networks_RH_ContC_pCun_2 | 14 | -72 | 40 |
| 353 | Right | Control | 17Networks_RH_ContC_pCun_3 | 6 | -64 | 44 |
| 354 | Right | Control | 17Networks_RH_ContC_pCun_4 | 8 | -50 | 44 |
| 355 | Right | Control | 17Networks_RH_ContC_pCun_5 | 8 | -72 | 52 |
| 356 | Right | Control | 17Networks_RH_ContC_Cingp_1 | 8 | -44 | 20 |
| 357 | Right | Control | 17Networks_RH_ContC_Cingp_2 | 6 | -28 | 28 |

## **Tab s3. Control Network Modal Controllability: Linear mixed effects models predicting executive functions cross-sectionally at Time 1 and Time 2**

| **Effects** | **Time 1 (N=170)** | | | | **Time 2 (N=262)** | | | |
| --- | --- | --- | --- | --- | --- | --- | --- | --- |
|  | **Estimate (SE)** | **T value** | **P value** | **95% CI [upper, lower]** | **Estimate (SE)** | **T value** | **P value** | **95% CI [upper, lower]** |
| Intercept | -0.53 (0.21) | -2.47 | 0.015 | [-0.95, -0.11] | -1.18 (0.18) | -6.53 | <0.0001 | [-1.53, -0.83] |
| Age | -0.10 (0.07) | -1.43 | 0.156 | [-0.24, 0.04] | -0.05 (0.05) | -0.96 | 0.340 | [-0.16, 0.05] |
| Ethnicity (White/Non-white) | 0.10 (0.23) | 0.44 | 0.661 | [-0.35, 0.56] | 0.52 (0.19) | 2.69 | 0.008 | [0.14, 0.89] |
| Health Status | -0.14 (0.06) | -2.14 | 0.034 | [-0.27, -0.01] | -0.13 (0.05) | -2.61 | 0.010 | [-0.23, -0.03] |
| Young Adult General Cognitive Ability | 0.33 (0.07) | 4.62 | <0.0001 | [0.19, 0.48] | 0.33 (0.06) | 5.82 | <0.0001 | [0.22, 0.44] |
| Control Network  Modal Controllability | 0.09 (0.06) | 1.46 | 0.146 | [-0.03, 0.22] | **0.12 (0.05)** | **2.43** | **0.016** | **[0.02, 0.22]** |

Note: Time 1 included 172 individuals, but after accounting for 2 missing data, Time 1 analyses included 170 individuals.

Time 2 included 267 individuals, but after accounting for 5 missing data, Time 2 analyses included 262 individuals.

## **Tab s4. Multiple Demand System Modal Controllability: Linear mixed effects models predicting executive functions cross-sectionally at Time 1 and Time 2**

| **Effects** | **Time 1 (N=170)** | | | | **Time 2 (N=261)** | | | |
| --- | --- | --- | --- | --- | --- | --- | --- | --- |
|  | **Estimate (SE)** | **T value** | **P value** | **95% CI [upper, lower]** | **Estimate (SE)** | **T value** | **P value** | **95% CI [upper, lower]** |
| Intercept | -0.55 (0.22) | -2.54 | 0.012 | [-0.97, -0.13] | -1.16 (0.18) | -6.44 | <0.0001 | [-1.51, -0.80] |
| Age | -0.11 (0.07) | -1.50 | 0.136 | [-0.25, 0.03] | -0.05 (0.05) | -0.91 | 0.364 | [-0.16, 0.06] |
| Ethnicity (White/Non-white) | 0.12 (0.23) | 0.53 | 0.598 | [-0.34, 0.58] | 0.50 (0.19) | 2.60 | 0.010 | [0.12, 0.87] |
| Health Status | -0.14 (0.06) | -2.21 | 0.028 | [-0.27, -0.02] | -0.14 (0.05) | -2.83 | 0.005 | [-0.24, -0.04] |
| Young Adult General Cognitive Ability | 0.33 (0.07) | 4.56 | <0.0001 | [0.19, 0.47] | 0.34 (0.06) | 5.92 | <0.0001 | [0.23, 0.45] |
| Multiple Demand System Modal Controllability | 0.10 (0.06) | 1.59 | 0.113 | [-0.02, 0.23] | **0.12 (0.05)** | **2.50** | **0.013** | **[0.03, 0.22]** |

Note: Time 1 included 172 individuals, but after accounting for 2 missing data, Time 1 analyses included 170 individuals.

Time 2 included 267 individuals, but after accounting for 5 missing data and 1 outlier, Time 2 analyses included 261 individuals.

## **Tab s5. Multiple Demand System Modal Controllability: Linear mixed effects models predicting changes in executive functions longitudinally from Time 1 to Time 2**

| **Effects** | **Time 1 & Time 2 (N=102)** | | | |
| --- | --- | --- | --- | --- |
|  | **Estimate (SE)** | **T value** | **P value** | **95% CI [upper, lower]** |
| Intercept | 0.30  (0.19) | 1.60 | 0.11 | [-0.07, 0.68] |
| Increase in Age  (from T1 to T2) | 0.04  (0.05) | 0.70 | 0.49 | [-0.07, 0.15] |
| Ethnicity (White/Non-white) | -0.02  (0.20) | -0.11 | 0.91 | [-0.42, 0.37] |
| Change in Health Status  (from T1 to T2) | 0.05  (0.05) | 0.91 | 0.37 | [-0.06, 0.16] |
| Young Adult General Cognitive Ability | 0.04  (0.06) | 0.65 | 0.52 | [-0.08, 0.15] |
| Change in Multiple Demand System Modal Controllability (from T1 to T2) | **0.14**  **(0.05)** | **2.53** | **0.01** | **[0.03, 0.24]** |

Note: Longitudinal data included 105 individuals, but after accounting for 3 missing data, longitudinal analyses included 102 individuals.

## **Tab s6. Control Network Modal Controllability: Linear mixed effects models predicting changes in executive functions longitudinally from Time 1 to Time 2**

| **Effects** | **Time 1 & Time 2 (N=102)** | | | |
| --- | --- | --- | --- | --- |
|  | **Estimate (SE)** | **T value** | **P value** | **95% CI [upper, lower]** |
| Intercept | 0.34  (0.19) | 1.76 | 0.08 | [-0.04, 0.72] |
| Increase in Age  (from T1 to T2) | 0.03  (0.05) | 0.58 | 0.56 | [-0.08, 0.14] |
| Ethnicity (White/Non-white) | -0.06  (0.20) | -0.32 | 0.75 | [-0.46, 0.34] |
| Change in Health Status  (from T1 to T2) | 0.05  (0.05) | 0.99 | 0.32 | [-0.05, 0.16] |
| Young Adult General Cognitive Ability | 0.03  (0.06) | 0.60 | 0.55 | [-0.08, 0.15] |
| Change in Control Network Modal Controllability (from T1 to T2) | 0.11  (0.05) | 1.99 | 0.05 | [0.00, 0.22] |

Note: Longitudinal data included 105 individuals, but after accounting for 3 missing data, longitudinal analyses included 102 individuals.

## **Tab s7. Control Network Modal Controllability X APOE genotype: Linear mixed effects models predicting executive functions cross-sectionally at Time 1 and Time 2**

| **Effects** | **Time 1 (N=170)** | | | | **Time 2 (N=233)** | | | |
| --- | --- | --- | --- | --- | --- | --- | --- | --- |
|  | **Estimate (SE)** | **T value** | **P value** | **95% CI [upper, lower]** | **Estimate (SE)** | **T value** | **P value** | **95% CI [upper, lower]** |
| Intercept | -0.54 (0.25) | -2.19 | 0.030 | [-1.02, -0.06] | -1.19  (0.22) | -5.35 | <0.0001 | [-1.62, -0.75] |
| Age | -0.10  (0.07) | -1.38 | 0.171 | [-0.24, 0.04] | -0.05 (0.06) | -0.86 | 0.391 | [-0.17, 0.06] |
| Ethnicity (White/Non-white) | 0.10 (0.23) | 0.44 | 0.663 | [-0.35, 0.56] | 0.46 (0.21) | 2.22 | 0.027 | [0.05, 0.87] |
| Health Status | -0.14 (0.07) | -2.19 | 0.030 | [-0.27, -0.02] | -0.12 (0.05) | -2.19 | 0.029 | [-0.22, -0.01] |
| Young Adult General Cognitive Ability | 0.34 (0.07) | 4.64 | <0.0001 | [0.19, 0.48] | 0.31 (0.06) | 5.21 | <0.0001 | [0.19, 0.44] |
| APOE Genotype (Positive/Negative) | 0.01  (0.16) | 0.09 | 0.928 | [-0.01, 0.52] | 0.12  (0.13) | 0.93 | 0.355 | [-0.13, 0.38] |
| Control Network  Modal Controllability | 0.25 (0.13) | 1.90 | 0.060 | [-0.30, 0.33] | 0.11 (0.12) | 0.98 | 0.330 | [-0.11, 0.34] |
| APOE X Modal Controllability | -0.21  (0.15) | -1.36 | 0.174 | [-0.50, 0.09] | 0.05  (0.13) | 0.36 | 0.722 | [-0.21, 0.30] |

Note: Time 1 included 172 individuals, but after accounting for 2 missing data, Time 1 analyses included 170 individuals.

Time 2 included 267 individuals, but after accounting for 5 missing data, as well as 29 missing data from APOE genotype, Time 2 analyses included 233 individuals.

## **Tab s8. Multiple Demand System Modal Controllability X APOE genotype: Linear mixed effects models predicting executive functions cross-sectionally at Time 1 and Time 2**

| **Effects** | **Time 1 (N=170)** | | | | **Time 2 (N=232)** | | | |
| --- | --- | --- | --- | --- | --- | --- | --- | --- |
|  | **Estimate (SE)** | **T value** | **P value** | **95% CI [upper, lower]** | **Estimate (SE)** | **T value** | **P value** | **95% CI [upper, lower]** |
| Intercept | -0.58 (0.25) | -2.34 | 0.021 | [-1.06, -0.09] | -1.16 (0.22) | -5.25 | <0.0001 | [-1.59, -0.73] |
| Age | -0.11 (0.07) | -1.46 | 0.148 | [-0.25, 0.04] | -0.05 (0.06) | -0.81 | 0.417 | [-0.16, 0.07] |
| Ethnicity (White/Non-white) | 0.13 (0.23) | 0.55 | 0.587 | [-0.33, 0.59] | 0.44 (0.21) | 2.11 | 0.036 | [0.03, 0.84] |
| Health Status | -0.14 (0.06) | -2.19 | 0.030 | [-0.27, -0.02] | -0.13 (0.05) | -2.50 | 0.013 | [-0.23, -0.03] |
| Young Adult General Cognitive Ability | 0.33 (0.07) | 4.59 | <0.0001 | [0.19, 0.47] | 0.32 (0.06) | 5.35 | <0.0001 | [0.20, 0.44] |
| APOE Genotype  (Positive/Negative) | 0.03  (0.16) | 0.20 | 0.845 | [-0.28, 0.35] | 0.13  (0.13) | 0.99 | 0.325 | [-0.13, 0.38] |
| Multiple Demand System Modal Controllability | 0.29 (0.14) | 2.09 | 0.038 | [0.02, 0.57] | 0.08  (0.13) | 0.62 | 0.536 | [-0.17, 0.33] |
| APOE X Modal Controllability | -0.24 (0.16) | -1.54 | 0.127 | [-0.54, 0.07] | 0.10  (0.14) | 0.68 | 0.494 | [-0.18, 0.37] |

Note: Time 1 included 172 individuals, but after accounting for 2 missing data, Time 1 analyses included 170 individuals.

Time 2 included 267 individuals, but after accounting for 5 missing data and 1 outlier, as well as 29 missing data from APOE genotype, Time 2 analyses included 232 individuals.

## **Tab s9. Control Network Modal Controllability X Young Adult General Cognitive Ability: Linear mixed effects models predicting executive functions cross-sectionally at Time 1 and Time 2**

| **Effects** | **Time 1 (N=170)** | | | | **Time 2 (N=262)** | | | |
| --- | --- | --- | --- | --- | --- | --- | --- | --- |
|  | **Estimate (SE)** | **T value** | **P value** | **95% CI [upper, lower]** | **Estimate (SE)** | **T value** | **P value** | **95% CI [upper, lower]** |
| Intercept | -0.59 (0.22) | -2.71 | 0.008 | [-1.01, -0.16] | -1.18 (0.18) | -6.52 | <0.0001 | [-1.53, -0.82] |
| Age | -0.11 (0.07) | -1.53 | 0.128 | [-0.25, 0.03] | -0.05 (0.05) | -0.96 | 0.337 | [-0.16, 0.05] |
| Ethnicity (White/Non-white) | 0.17 (0.23) | 0.71 | 0.481 | [-0.29, 0.62] | 0.52 (0.19) | 2.70 | 0.008 | [0.14, 0.90] |
| Health Status | -0.13 (0.06) | -2.06 | 0.041 | [-0.26, -0.01] | -0.13 (0.05) | -2.57 | 0.011 | [-0.23, -0.03] |
| Young Adult GCA | 0.31 (0.07) | 4.29 | <0.0001 | [0.17, 0.46] | 0.33  (0.06) | 5.80 | <0.0001 | [0.22, 0.44] |
| Control Network  Modal Controllability | 0.10 (0.06) | 1.54 | 0.125 | [-0.03, 0.22] | 0.12 (0.05) | 2.41 | 0.016 | [0.02, 0.22] |
| Young Adult GCA X Modal Controllability | -0.10  (0.06) | -1.62 | 0.107 | [-0.22, 0.02] | -0.02  (0.05) | -0.39 | 0.693 | [-0.12, 0.08] |

Note: GCA=General Cognitive Ability

Time 1 included 172 individuals, but after accounting for 2 missing data, Time 1 analyses included 170 individuals.

Time 2 included 267 individuals, but after accounting for 5 missing data, Time 2 analyses included 262 individuals.

## **Tab s10. Multiple Demand System Modal Controllability X Young Adult General Cognitive Ability: Linear mixed effects models predicting executive functions cross-sectionally at Time 1 and Time 2**

| **Effects** | **Time 1 (N=170)** | | | | **Time 2 (N=261)** | | | |
| --- | --- | --- | --- | --- | --- | --- | --- | --- |
|  | **Estimate (SE)** | **T value** | **P value** | **95% CI [upper, lower]** | **Estimate (SE)** | **T value** | **P value** | **95% CI [upper, lower]** |
| Intercept | -0.57 (0.22) | -2.58 | 0.011 | [-1.00, -0.14] | -1.15 (0.18) | -6.38 | <0.0001 | [-1.50, -0.80] |
| Age | -0.11 (0.07) | -1.52 | 0.132 | [-0.25, 0.03] | -0.05 (0.05) | -0.91 | 0.366 | [-0.16, 0.06] |
| Ethnicity (White/Non-white) | 0.15 (0.24) | 0.62 | 0.533 | [-0.32, 0.62] | 0.49 (0.19) | 2.58 | 0.011 | [0.12, 0.87] |
| Health Status | -0.14 (0.06) | -2.19 | 0.030 | [-0.27, -0.01] | -0.14 (0.05) | -2.81 | 0.005 | [-0.24, -0.04] |
| Young Adult GCA | 0.32 (0.07) | 4.30 | <0.0001 | [0.17, 0.47] | 0.34 (0.06) | 5.91 | <0.0001 | [0.22, 0.45] |
| Multiple Demand System Modal Controllability | 0.10 (0.06) | 1.61 | 0.109 | [-0.02, 0.23] | 0.12 (0.05) | 2.31 | 0.022 | [0.02, 0.22] |
| Young Adult GCA X Modal Controllability | -0.03  (0.07) | -0.49 | 0.623 | [-0.16, 0.10] | -0.03  (0.05) | -0.56 | 0.573 | [-0.13, 0.07] |

Note: GCA=General Cognitive Ability

Time 1 included 172 individuals, but after accounting for 2 missing data, Time 1 analyses included 170 individuals.

Time 2 included 267 individuals, but after accounting for 5 missing data and 1 outlier, Time 2 analyses included 261 individuals.

## **Tab s11. Correlations among Variables at Time 1 in Cross-sectional Participants**

|  | Executive Function | | Age | Health Status | APOE Genotype (1=Positive, 0=Negative) | Young Adult GCA | Multiple Demand System MC |
| --- | --- | --- | --- | --- | --- | --- | --- |
| Age | | -0.08  p=0.2855 |  |  |  |  |  |
| Health Status | | -0.21  p=0.0048 | 0.02  p=7875 |  |  |  |  |
| APOE Genotype (1=Positive, 0=Negative) | | -0.04  p=0.5622 | 0.10  p=0.1988 | -0.07  p=0.3437 |  |  |  |
| Young Adult GCA | | 0.36  p<.0001 | 0.13  p=0.0984 | -0.11  p=0.1509 | -0.03  p=0.6948 |  |  |
| Multiple Demand System MC | | 0.09  p=0.2273 | 0.12  p=0.1326 | -0.08  p=0.2882 | 0.02  p=0.7500 | 0.00  p=0.9755 |  |
| Control Network MC | | 0.08  p=0.2715 | 0.07  p=0.3408 | -0.12  p=0.1223 | -0.01  p=0.8613 | -0.03  p=0.752 | 0.86  p<.0001 |

Note: GCA=General Cognitive Ability, MC=Modal Controllability.

## **Tab s12. Correlations among Variables at Time 2 in Cross-sectional Participants**

|  | Executive Function | | Age | Health Status | APOE Genotype (1=Positive, 0=Negative) | Young Adult GCA | Multiple Demand System MC |
| --- | --- | --- | --- | --- | --- | --- | --- |
| Age | | 0.00  p=0.9750 |  |  |  |  |  |
| Health Status | | -0.15  p=0.0141 | 0.05  p=0.3793 |  |  |  |  |
| APOE Genotype (1=Positive, 0=Negative) | | -0.06  p=0.3286 | 0.04  p=0.5850 | -0.03  p=0.6442 |  |  |  |
| Young Adult GCA | | 0.45  p<.0001 | 0.14  p=0.02 | -0.06  p=0.3793 | -0.01  p=0.9108 |  |  |
| Multiple Demand System MC | | 0.15  p=0.0115 | -0.02  p=0.6915 | 0.09  p=0.1636 | 0.11  p=0.0770 | 0.10  p=0.1193 |  |
| Control Network MC | | 0.12  p=0.0426 | -0.04  p=0.4817 | 0.06  p=0.3255 | 0.10  p=0.1209 | 0.09  p=0.1684 | 0.83  p<.0001 |

Note: GCA=General Cognitive Ability, MC=Modal Controllability

**References**

1 Golden CJ. Stroop Color and Word Test*.* Multi-Health Systems; 2003.

2 Delis DC, Kaplan E, Kramer JH. Delis-Kaplan Executive Function System (D-KEFS)*.* Psychological Corporation: San Antonio, TX; 2001.

3 Wechsler D. Wechsler Memory Scale (WMS-III)*.* Psychological Corporation: San Antonio, TX; 1997.

4 Daneman M, Merikle PM. Working memory and language comprehension: A meta-analysis. Psychonomic Bulletin. 1980;3:422-33.

5 Assem M, Glasser MF, Van Essen DC, Duncan J. A Domain-General Cognitive Core Defined in Multimodally Parcellated Human Cortex. Cereb Cortex. 2020;30(8):4361-80.
